# Can reminders improve adherence to regular physical activity and exercise recommendations in people over 60 years old? A randomized controlled study

**DOI:** 10.1007/s00508-020-01699-6

**Published:** 2020-06-26

**Authors:** Gudrun Wolner-Strohmeyer, Mohammad Keilani, Bruno Mähr, Eva Morawetz, Andrej Zdravkovic, Barbara Wagner, Stefano Palma, Michael Mickel, Galateja Jordakieva, Richard Crevenna

**Affiliations:** 1 Wien Hauptstelle, Versicherungsanstalt öffentlich Bediensteter, Eisenbahnen und Bergbau, Vienna, Austria; 2 Department of Physical Medicine, Rehabilitation and Occupational Medicine, Medical University of Vienna, Währinger Gürtel 18–20, 1090 Vienna, Austria; 3 Therapiezentrum Rosalienhof, Versicherungsanstalt öffentlich Bediensteter, Eisenbahnen und Bergbau, Bad Tatzmannsdorf, Austria

**Keywords:** Regular physical activity, Reminders, Austrian Insurance Fund for Civil or Public Servants, Pensioners

## Abstract

**Purpose:**

The purpose of the study was to investigate whether additional reminders could enhance adherence to a 12-week program consisting of regular physical activity.

**Methods:**

The study collective consisted of pensioners insured with the Austrian Insurance Fund for Civil or Public Servants. They were made aware of our program through the public service union. The subjects were randomized to an intervention group (group A) that received reminders and to a control group (group B) that did not receive such notifications. Adherence to physical activity was assessed by the use of diaries.

**Results:**

Group A performed 96 min more moderate intensity regular physical activity per week than group B (group A median 269 min, r = 0–1560 min; group B median 173 min, r = 0–2700 min). The Mann–Whitney *U*-test showed no significant differences (*p* = 0.080) between the study groups.

There was no difference in muscle strengthening activity (group A: median: 2, r = 0–13 sessions; group B: median: 2, r = 0–20 sessions).

**Conclusion:**

The major positive observation was that both the experimental and control group participants exceeded the recommended level of physical activity. Nevertheless, there were some differences concerning the minutes of physical activity performed in favor of the intervention group.

## Introduction

Life expectancy is rising in Europe continuously [1] but at the same time, there seems to be decline of healthy life years in Europe [1].

Participating in regular physical activity and exercise programs improves the level of health and state of well-being in older adults [2].

Regular physical activity and exercise are performed in accordance with international guidelines [3, 4]. Despite the basic knowledge about the necessity of such programs and the associated changes in behavior, many older people seem to have low adherence to regular physical activity and exercise interventions [2].

The primary purpose of the study was to investigate whether additional reminders could enhance adherence to a 12-week program consisting of regular physical activity.

The secondary objective was the evaluation of effects on anthropometric parameters, blood pressure, heart rate, endurance ratio, and muscle strength at the beginning and end of medical examinations.

## Patients, material and methods

The present randomized controlled study was approved by the ethics committee of the Medical University of Vienna (EK-Nr: 2296/2016). The study collective consisted of pensioners (at least 60 years old) insured with the Austrian Insurance Fund for Civil or Public Servants (formerly Versicherungsanstalt öffentlich Bediensteter, BVA; since January 2020: Versicherungsanstalt öffentlich Bediensteter, Eisenbahnen und Bergbau, BVAEB). They were made aware of our program through the public service union. Written informed consent was obtained from all the participants prior to enrolment. The exclusion criteria were according to international recommendations for regular physical activity and exercise as published before [2].

The participants were contacted via postal mailings and an email distribution list, which were provided by the Federal Representation of Pensioners of the Public Service Union of Austria. A total of around 10,000 pensioners were invited to participate in the study. More than 200 were interested. For organizational reasons (e.g. space and time capacity), the first 157 people were consecutively registered by telephone for a kick-off meeting. In addition, there was a waiting list with further participants for a possible follow-up project. In an information event, interested participants received lectures about regular physical activity according to established guidelines, nutrition, and mental health [2–4].

During and after the presentations, interested persons had the opportunity to register in a list for participation in the study. All participants gave their informed consent prior to their inclusion in the study. In the following few days, the participants were contacted to arrange an appointment for the baseline examination (T1). These examinations took place in March 2017.

The baseline examination at T1 included a survey of demographic data (age and sex), a comprehensive history taking and a physical examination including an electrocardiogram.

Participants with suspicious findings were sent for further examinations. For those persons who had no exclusion criteria, further assessments were performed.

The randomization was carried out after T1. The subjects were randomized to an intervention group (group A) that received reminders, and to a control group (group B) that did not receive notifications. In case of married couples or persons living in partnership, both partners were assigned to the same group (group A or group B) in order to minimize bias.

Group A received reminder messages twice a week via emails or short message service (SMS). The content of these messages was always identical, calling upon the participants to regularly perform their recommended physical activity. All but two subjects chose to be reminded by email.

The program consisted of regular physical activity according to established guidelines [2–4].

The follow-up examination (T2) took place at the end of the program. For group A this was 2.5 ± 0.9 weeks, for group B 12.3 ± 0.7 weeks after T1.

## Assessment

We assessed the adherence to physical activity by the use of diaries. They contained detailed information about the duration of the moderate intensity as well as of the vigorous intensity physical activity performed that was recorded in minutes per day.

Anthropometric data (body mass index, waist circumference, blood pressure and pulse) were assessed.

Blood pressure (mmHG) and pulse (per min) were measured at the upper arm with a boso medicus PC (Bosch + Sohn GmbH u. Co. KG, Jungingen, Germany) [5].

Handgrip strength (lbs) was measured using a Jamar® dynamometer (Lafayette Instrument Copany, Lafayette, IN, USA) [6].

Endurance capacity was assessed by the use of the 6‑minute walk test (6MWT, m) [7].

## Statistical analysis

Descriptive statistics were calculated for the outcome variables at T1 and T2 (pre/post).

Kolmogorov-Smirnov and Shapiro-Wilk tests were used to assess the normality of the data.

Because the distribution of the reported total minutes of moderate intensity physical activity showed outliers, for statistical testing the Mann–Whitney *U*-test was used for further analysis. *P* values ≤0.05 were considered to be statistically significant. All computations have been performed using the IBM SPSS Statistics Version 22.0.0.0.

## Results

Of the 157 subjects who had registered for the program, 133 finally took part in the kick-off meeting and 126 of them agreed to participate in the baseline examination (T1), in which 121 persons took part. Of these, 26 subjects were excluded from the study according to our exclusion criteria and further examinations had to be performed (Table 1). The remaining 95 participants were randomized in group A (*n* = 47) and group B (*n* = 48). The reasons for drop-out are presented in Table 1.

**Table 1:** Reasons of exclusion or drop-out of the study population

Reason for exclusion/drop out	Number
*Excluded during T1*	*n* = 26
Serious cardiovascular disease	*n* = 18
Pneumonia	*n* = 1
Serious urinary tract infection	*n* = 1
Femoral fracture	*n* = 1
Serious ocular disease	*n* = 1
Inguinal hernia	*n* = 1
Serious vertigo	*n* = 1
Metastatic prostate cancer during radiation therapy	*n* = 1
Serious hepatic disease	*n* = 1
*Dropped out between T1 and T2*	*n* = 11
Serious cardiovascular disease	*n* = 2
Middle foot fracture	*n* = 1
Serious orchitis	*n* = 1
Serious sciatica	*n* = 1
Familiar reasons	*n* = 2
No longer available	*n* = 1
Unsecure about data protection	*n* = 1
Thought that she was excluded from the study	*n* = 1
Not specified	*n* = 1
*Dropped out at T2*	*n* = 11
Did not fill in their diaries	*n* = 6
Serious injury	*n* = 2
Serious sciatica	*n* = 1
Serious laryngitis	*n* = 1
Eye surgery	*n* = 1

*T1* baseline examination**, ***T2* follow-up examination

We evaluated in the remaining participants (*n* = 73, male:female = 28:45, age: 61–82 years) the data at T1 as well as T2. Of the participants 27 suffered from musculoskeletal diseases, 18 from cardiovascular diseases, and 5 from cancer but were not severe and did not lead to exclusion from the study.

Participants in group A performed 96 min more moderate intensity regular physical activity per week than group B (Table 2). The Mann–Whitney *U*-test showed that there was no significant difference between both study groups (Table 2). At the vigorous intensity there was no notable difference (Table 2).

**Table 2:** Descriptive statistics of adherence to regular physical activity (*n* = 73, male:female = 28:45, age: 61–82 years). The reported total minutes of moderate physical activity were analyzed by the Mann–Whitney *U*-test

Variable	A	B	Difference
*MPA (min)*	269 (r = 0–1560)	173 (r = 0–2700)	+96 min (*p* = 0.080)
*VPA (min)*	61 ± 90 (r = 0–1275)	67 ± 76 (r = 0–535)	−6 min (not notable)
*MSA (sessions)*	3 ± 2 (r = 0–13)	3 ± 3 (r = 0–20)	No difference

*A* interventional group, *B* control group, *MPA* moderate intensity physical activity, *VPA* vigorous intensity physical activity, *MSA* muscle strengthening activity

Concerning muscle-strengthening activity there was no difference (Table 2).

Systolic blood pressure (group A : T1 = 147 ± 19 mm Hg, T2 = 128 ± 13 mm Hg = −12%; group B : T1 = 145 ± 17 mm Hg, T2 = 131 ± 17 mm Hg = −10%) as well as diastolic blood pressure (group A : T1 = 86 ± 11 mm Hg, T2 = 82 ± 8 mm Hg = −3%; group B : T1 = 85 ± 9 mm Hg, T2 = 80 ± 8 mm Hg = −5%) were not different between the groups. Nonetheless, blood pressure was lowered in both groups by the end of the study.

The measurements of Body Mass Index (BMI), waist circumference, 6MWT as well as handgrip strength showed no notable changes between T1 and T2 in both groups (A and B).

## Discussion

Poor adherence diminishes the clinical benefits of regular physical activity [2].

The results of the present study showed that our participants achieved or even overachieved the goals of the guidelines for regular physical activity (Table 1). The results could be due to the fact that subjects insured with the BVA usually have higher educational and professional qualification levels. According to the literature, factors associated with adherence to exercise in older adults include higher socioeconomic and educational levels [2]; however, we did not assess these parameters, which is a limitation of the study.

Regular reminders might improve adherence; however, this trend was not significant in our study. Therefore, short-term intensive information as in the present study (including the provision of written documents) might be sufficient to maintain adherence over a relatively short period. In comparison, Cole-Lewis and Kershaw also reported short-term effects of regular reminders on adherence to preventive and management strategies [8]. In another study by De Leon et al. [9] positive results for short-term behavioral changes were described, while Mbuagbaw et al. considered SMS messages as an effective tool for lifestyle changes [10].

Eakin et al. underlined the benefits of telephone interventions concerning regular physical activity and dietary behavior [11].

A study duration between 6–12 months is recommended for lifestyle interventions to achieve notable benefits [12, 13]. Therefore, the 12-week term of our program is a limitation of our study. Nevertheless, the focus of those studies was not primarily the measurement of adherence to exercise [12, 13]. Further studies with larger samples and longer observation periods would be necessary to clarify whether regular reminders efficiently support adherence to regular physical activity.

The setting of the present study has some advantages as well as disadvantages. An advantage of this multicomponent exercise program might be that the program can be performed all year round and in a variety of settings, including school, community, family and home, and primary care. Furthermore, participants could plan their activities individually and decided themselves when to complete the recommended physical activity.

At the same time all the advantages can also be perceived as potential disadvantages. Some of the respondents, for example, noticed at the time of the follow-up (T2) examination that a group setting would have been more motivational or that more intensive support through meetings of the participants would have increased adherence. On the other hand, some other participants found their independence very pleasant.

In the present study, blood pressure was lowered in both groups by the end of the study, which is very positive and should encourage the study participants to continue regular physical activity.

In contrast to blood pressure, the parameter heart rate did not notably change during the study duration. This could be due that the aim of our study was not to perform systematic aerobic exercise.

The results of the 6MWT in both study groups (A + B) were remarkably better than sex and age related reference values [14, 15]. This result indicates that the study participants were physically active even before inclusion in the study, which might be a selection bias.

The outcome variables waist circumference as well as body mass index did not show any notable differences between both groups. Nevertheless, in view of the short observation period and the objective of the present study, namely to primarily investigate adherence and not to change body composition, these results are not surprising.

The present study has some limitations. The short study duration is one limitation. Additionally, we did not perform a power analysis. Further limitations are the relatively high drop-out rate of 21% during the study and the high exclusion rate (= 26) at the beginning of this study. The high drop-out rate could possibly be explained by the fact that the study collective was not preselected due to our study design. On the other hand, the drop-out as well as exclusion rates showed that medical checks before the start of such interventions are essential for patient safety.

The medical consultation during the initial examination was also important. This made it possible to detect contraindications for participation in the study among the older group of participants. The medical consultation could represent a valuable health policy strategy to effectively shape health promotion in the population.

The on-going coronavirus disease (COVID-19) outbreak in all continents has become the world’s leading health headline and is causing major public concern [16]. At the moment, staying at home is a safe measure. Therefore, there is a strong rationale for continuing regular physical activity at home to stay healthy in the current environment. Regular physical activity at home by performing various safe, simple, and easily implementable exercises is suitable to avoid coronavirus infection and maintain muscle strength as well as muscle endurance [16].

Adherence to recommendations for regular physical activity at the moment a challenging issue [17]. In the present study, the participants were informed during a meeting (long before COVID-19). At the moment, meetings must be avoided due to COVID 19. Therefore, alternative as well as safe strategies (online platforms with individual passwords, SMS messages, email) are needed to provide sufficient information.

## Conclusion

The major positive observation was the fact that both in the experimental and the control group participants exceeded the recommended level of physical activity. Nevertheless, there were some differences concerning the minutes of physical activity performed in favor of the intervention group.

Further studies with larger study samples and longer observation periods are necessary to investigate whether regular reminders can significantly improve adherence to regular physical activity in older people.
